# Individual differences predict endorsement of water resilience

**DOI:** 10.1038/s41598-020-62896-x

**Published:** 2020-04-06

**Authors:** Julia Baird, Gillian Dale, Sherman Farhad

**Affiliations:** 1 Environmental Sustainability Research Centre, Brock University, Ontario, Canada; 2 Department of Geography and Tourism Studies, Brock University, Ontario, Canada

**Keywords:** Psychology and behaviour, Sustainability

## Abstract

In the epoch of the Anthropocene change, complexity, and uncertainty create a demand for new systems of water management and governance. One such management model that is rapidly gaining traction amongst both scholars and practitioners is the concept of *water resilience*. Although increasing attention has been paid to the overarching theoretical and applied issues surrounding water resilience, few have examined individual attitudes and perceptions towards this concept. In this paper, we examine to what extent individuals endorse – that is, agree with and see the importance of using - social-ecological resilience as a framework for management and governance of water resources. We approach the problem and promise of water governance in this way because individuals’ mindsets (and shifts in mindsets) offers one of the most effective leverage points for larger system change. To explore water resilience endorsement, we developed a scale (i.e., a water resilience scale) that was designed to capture individual endorsement of each of the seven principles of social-ecological water resilience. Three additional sets of questionnaires were also used to examine whether individual characteristics (i.e., demographics, psychological factors, and environmental attitudes) predict water resilience endorsement. Overall, there was considerable societal endorsement of water resilience. However, the degree to which individuals endorsed the concept of water resilience differed as a function of demographics, psychological characteristics, and attitudes toward the environment. Future research should examine the nuances of endorsement and consider targeted approaches to influence endorsement levels by using the predictor variables as a basis for engaging and shifting mindsets.

## Introduction

The Anthropocene^1–3^ is a theoretical new geological epoch characterized by significant human-produced global changes both in terms of magnitude and speed. Water is critical to sustainability in this era, and “ensuring availability and sustainable management of water and sanitation for all” is one of the primary goals set by the United Nations in the 2030 Agenda for sustainable development^4^. Given the uncertainty and complexity associated with this transitional period, there are increasing calls for new water governance and management approaches that consider the whole social-ecological system^5^ and that are based on complex systems thinking^6,7^. One such approach is that of social-ecological resilience - the capacity of a social-ecological system to cope with changes while maintaining its identity and critical functions^8–11^. Embracing change lies at the heart of resilience thinking^11^, and this concept provides a pathway towards sustainability in a complex and uncertain world where change is inevitable. As such, it is critical to implement governance and management approaches that align with a social-ecological resilience perspective^5,8,12–16^, and to understand the challenges and opportunities for implementing these approaches.

A challenge associated with the implementation of social-ecological resilience in water governance (‘water resilience’ hereafter) lies in society’s endorsement of a water resilience approach. We define endorsement here as a combination of agreement with, and belief in the importance of, an approach. It is important to understand what motivates individuals to endorse water resilience because acceptance and endorsement of a new approach at the individual level enables its further legitimization and communication in broader society^17–19^, thereby leading to transformation in governance (including new sets of rules, values, and practices^20^). We approach these questions from the perspective of individual endorsement of water resilience, recognizing that individuals can effect change at a much broader level in social-ecological systems, and that individual traits (or ‘inner worlds’) such as empathy e.g.^21^, spirituality e.g.^22^, and political values e.g.^23^ can be used as a mechanism for building water resilience endorsement^24,25^. However, to develop effective mechanisms for building water resilience endorsement (and ultimately create the potential for water governance transformations), we first need to understand the individual characteristics that may be associated with varying levels of endorsement. Accordingly, the aim of our study is to examine individual differences in water resilience endorsement. ‘Water resilience’ is still a relatively new term in both scholarly and non-scholarly fields, and diverse approaches are being developed, stemming from varying conceptualizations. In the academic context, the water resilience literature is divided into different types, such as engineering, ecological, social-ecological, community, institutional, and disaster. Water resilience has also gained attraction outside of scholarship and has been increasingly incorporated into water policy language across regions and sectors. Although different approaches are available (such as critical infrastructure security, risk management, and adaptation technologies among others), a holistic perspective is also being employed by a few organizations (e.g., FAO-World Bank, IRGC, OECD, and The Rockefeller Foundation)^26–29^. We acknowledge these differences and here focus on a definition consistent with a social-ecological systems perspective: “the capacity to adapt or transform in the face of change in social-ecological systems, particularly unexpected change, in ways that continue to support human wellbeing”^30^ with a specific focus on water resources.

Biggs *et al*.^31,32^ have identified seven generic principles for enhancing social-ecological resilience, which could be applied in diverse governance and management contexts, including water-focused social-ecological systems: (P1) maintain diversity and redundancy, (P2) manage connectivity, (P3) manage slow variables and feedbacks, (P4) foster an understanding of social-ecological systems as complex adaptive systems, (P5) encourage learning and experimentation, (P6) broaden participation, and (P7) promote polycentric governance systems. We apply these principles in the present study as a mechanism to unpack water resilience.

Meeting the challenges of the Anthropocene requires “unprecedented transformative solutions for sustainability with a careful consideration of resilience in their implementation”^33^. Implementing a water resilience approach is challenging not least because it requires endorsement from society. Deeper insights into the perceptions of individuals in society regarding a water resilience-based approach in the management of water resources are needed, and understanding the individual differences that are related to greater or lesser endorsement is critical for moving forward to improve water resilience endorsement. The purpose of this study is therefore to understand to what extent individuals in society (in this case, Canadian and United States residents) endorse – that is, agree with and believe in the importance of using - water resilience as a framework for management and governance of water resources. The specific objectives of this research are to: 1) Develop and test a “water resilience scale” for assessing endorsement of water resilience principles; 2) Describe the level of water resilience endorsement of the Canadian and US public; and, 3) Identify individual differences that predict levels of water resilience endorsement.

## Method

### Participants

A total of 562 Canadian (N = 268) and American (N = 294) individuals participated in this study via Amazon Mechanical Turk (MTurk; see Paolacci & Chandler^34^ on the quality of MTurk samples) in 2018. A breakdown of the demographics of this sample can be found in Supplementary Table S1. The study took approximately 30 minutes to complete, and participants received $4.50 USD as compensation. In order to be eligible to participate, participants had to be located in Canada or the United States, fluent in English, and had to be at least 18 years of age. All subjects provided written consent prior to participating. This study was approved by the Human Research Ethics Board at Brock University, and conducted in accordance with Tri-Council ethical guidelines

### Stimuli and design

Eligible participants were asked to complete a 30-minute survey, presented on the Qualtrics platform and accessible via MTurk, regarding their views on the environment. The survey consisted of 4 sections (see below for details): 1) a demographics questionnaire, 2) 5 psychological measures, 3) questions about the local environment and environmental change, and 4) a questionnaire designed to assess their level of agreement with the principles of water resilience. All participants completed the questions in the same order, and after completion of the survey were debriefed and compensated.

#### Demographics

The demographics questionnaire was designed to collect the following personal information: age, sex, country, province/state, years in current location, highest level of education, employment status, household income, marital status, number of children, religious importance and attendance, and political affiliation. These questions were based off of similar questions from the United States Census and the Canadian Census to ensure that they were culturally appropriate and clear.

### Psychological factors

#### Mini-IPIP personality scale

This 20-item questionnaire, adapted from Donnellan *et al*.^35^, was designed to assess all five dimensions of the Big-5 personality model (i.e., openness to experience, conscientiousness, extraversion, agreeableness, and neuroticism). Each question was answered using a 7-point Likert scale, based on the degree to which the participant felt each item in the questionnaire corresponded to their own behaviours (1 = very inaccurate; 7 = very accurate). The average rating for each of the 5 personality factors was calculated, with higher scores reflecting higher levels of a given personality trait.

#### Brief locus of control questionnaire

The brief locus of control questionnaire assesses the degree to which individuals believe they have control over the events that occur in their life^36^. Participants were asked to rate whether or not they agreed with 10 statements using a 7-point Likert scale (1 = “I do not agree at all”; 7 = “I fully agree”). Half of the statements assessed internal locus of control (i.e., the belief that outcomes are due to one’s own behaviour, efforts, or abilities; e.g., “How my life takes course is entirely dependent on me”), and the other half assessed external locus of control (i.e., the belief that outcomes are due to external factors beyond one’s control; e.g., “My achievements are mainly due to destiny and luck”). An average internal locus of control and an average external locus of control score was calculated for each participant, with higher scores indicating higher levels of locus of control.

#### Adapted empathy scale

This 19-item questionnaire was adapted from the Adult Impulsivity scale^37^. The full version of this questionnaire assesses empathy, as well as impulsivity and risk-taking, whereas the version used here only included the empathy items. Participants were asked to indicate “yes” or “no” to the 19 questions (e.g., “Would you feel sorry for a lonely stranger?”). An empathy score was generated by summing the number of “yes” scores (or “no” scores in the case of a reverse-keyed item) for a total score out of 19 (higher scores = higher levels of empathy).

#### New general self-efficacy scale

This questionnaire assesses the belief in one’s own capacity to succeed or accomplish a goal/task^38^. Participants used a 5-point Likert scale to rate 8 statements (e.g., “I will be able to achieve most of the goals that I have set for myself”) based on the degree to which they felt each statement described to their own behaviours (1 = very inaccurate; 5 = very accurate). A self-efficacy score was calculated by averaging the ratings across all 8 items, for a possible maximum score of 5 (higher scores = higher levels of self-efficacy).

#### Resistance to change questionnaire

This questionnaire, adapted from Oreg^39^, assesses beliefs and attitudes toward making changes (e.g., “When things don’t go according to plans, it stresses me out”). Participants were asked to rate how accurately 17 statements described their feelings toward change by using a -point Likert scale (1 = “Strongly Disagree”; 6 = “Strongly Agree”). An average resistance to change score was calculated for each participant, with higher scores indicating greater resistance to change.

### Environment questions

#### Environmental futures

This scale was adapted from Gifford *et al*.^40^, and is used to assess individual perceptions of both the current and future state of the environment. For the “current” section, participants were asked to rate the current condition of various aspects of the environment (e.g., the availability of fresh drinking water; biodiversity; the state of fisheries; the management of natural disasters) on a scale from 1 (very bad) to 5 (very good) for a) their city/town, and b) their country. For the “future” section, participants were asked to predict what the comparative state of the environment will be in 25 years on a scale from 1 (much worse than now) to 5 (much better than now) for a) their city/town, and b) their country.

A “local optimism” score was calculated for each participant by taking the difference between the current and future ratings (across all questions) for the participant’s city/town (average future city/town rating –average current city/town rating). Similarly, a “national optimism” score was calculated by taking the difference between current and future estimates of the state of the environment in one’s country (average future country rating –average current country rating). For both the local and national optimism scores, positive scores indicate greater optimism that the environmental condition will improve in the future, whereas negative scores indicate a belief that conditions will worsen over time.

#### Other

In addition to the environmental futures scale, we asked participants a series of water-related questions including the importance/meaning of their local water bodies and their attachment to said water sources, how often they visit their local water bodies, and the water-related disasters (e.g., floods, drought, invasive species, etc.) that have struck their region in the past 4 years. We also asked participants two questions regarding their beliefs on climate change (i.e., whether or not it is serious, and whether they believe that it is driven by human actions), and two questions to assess whether or not the participant would be willing to make a change in their personal life, or would be comfortable with society making a change, that would benefit the environment.

#### Water resilience questionnaire

A series of questions were developed to assess individual endorsement of the 7 principles of water resilience (See Supplementary Method for our development of a water resilience scale). The questions were broad in their scope (using the term ‘water’ but not defining specific contexts or situations). This questionnaire was extensively piloted in order to ensure broad understanding of the language used by a lay audience. Half of the questions asked participants to rate the importance of the seven principles relating to resilience (e.g., “How important is it to you that many perspectives are included in decision making about water?”), and half asked participants to indicate the extent to which they agreed with a number of statements relating to resilience principles (e.g., “Decision makers should focus on immediate threats to our water, and not spend a lot of time on monitoring for longer term potential problems.”). Half of the questions were reverse scored. An average score for each of the 7 principles was calculated, with higher scores indicating higher levels of endorsement of a given principles. An exploratory factor analysis (PCA) with varimax rotation and Kaiser normalization indicated that mean endorsement ratings for all 7 principles loaded onto a single factor, suggesting that they represent a common underlying construct. As such, we also calculated an overall endorsement score by taking the average of scores across all 7 principles. The Cronbach’s alpha for the resilience questionnaire was 0.769, indicating acceptable internal consistency reliability.

## Results

**Figure 1:**
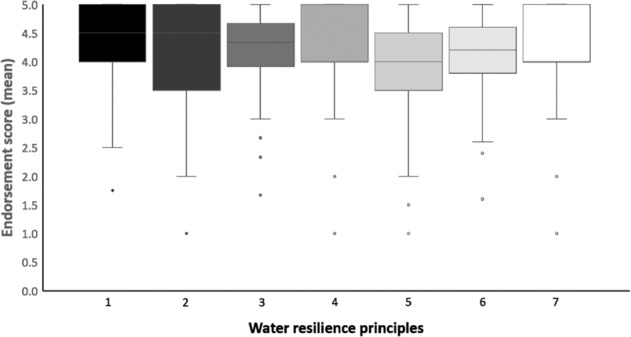
Boxplot showing median scores, distribution, and minimum/maximum scores for each of the 7 resilience principles.

The means and SDs for the demographic measures, the psychological measures, and the environmental measures can be found in Supplementary Tables S1–S3, respectively. All data were normally distributed, and unless otherwise specified all statistical tests were two-tailed. For the resilience scale, Fig. 1 illustrates the median score for each of the 7 principles and the distribution of scores across participants. Overall resilience endorsement scores ranged from 2.61 to 5 (out of a maximum possible score of 5), with a mean overall score of 4.19 (*SD* = 0.47), indicating that participants as a whole were accepting of resilience principles.

### Resilience endorsement and individual differences

Next, we conducted a correlational analysis to examine the relationships between overall resilience endorsement and our measures of demographics, psychological factors, and environmental factors. Given the number of relationships that were examined, only significant results are reported here.

#### Demographics

For the demographic variables there were several small, but significant, correlations with overall resilience endorsement. There was a significant correlation between resilience endorsement and sex (*r* = −0.107, *N* = 562), age (*r* = 0.183, *N* = 559), and marital status (*r* = 0.133, *N* = 562), such that being female, older, and married was associated with higher endorsement of resilience. Both religious attendance (*r* = −0.158, *N* = 562), and religious importance (*r* = −0.121, *N* = 562) were also significantly negatively correlated with resilience endorsement, such that attending fewer religious services, and assigning less importance to religion, were both associated with higher resilience endorsement. Finally, political party was significantly associated with resilience endorsement (*r* = 0.185, *N* = 562), such that being affiliated with the democrat/liberal party, as opposed to the republican/conservative party, was associated with higher resilience endorsement. No other correlations reached the level of significance.

#### Psychological factors

Nearly all of the psychological factors were significantly associated with resilience endorsement. For the personality measures, overall resilience endorsement was significantly correlated with openness to experience (*r* = 0.395, *N* = 562), conscientiousness (*r* = 0.197, *N* = 562), agreeableness (*r* = 0.381, *N* = 562), and neuroticism (*r* = −0.157, *N* = 562), such that higher levels of each trait (and lower levels of neuroticism) were associated with higher endorsement. There was no relationship between resilience endorsement and extraversion (*r* = 0.04, *N* = 562), however. Both internal (*r* = 0.221, *N* = 562) and external (*r* = −0.217, *N* = 562) locus of control were significantly correlated with resilience endorsement, such that having a higher internal locus of control was associated with greater endorsement of resilience, whereas having a higher external locus of control was associated with less endorsement. There was also a strong correlation between resilience endorsement and empathy (*r* = 0.303, *N* = 562), such that higher innate levels of empathy were associated with greater endorsement. Similarly, there was a moderate correlation between resilience endorsement and self-efficacy (*r* = 0.255, *N* = 562), such that a greater belief in one’s ability to succeed was associated with greater resilience endorsement. Finally, resilience endorsement and resistance to change were significantly negatively correlated (*r* = −0.188, *N* = 562), such that individuals who were less resistant to change were more likely to endorse resilience principles.

#### Environmental factors

Overall resilience endorsement was significantly correlated with each of our environmental factors. Both local (*r* = −0.180, *N* = 562) and national (*r* = −0.149, *N* = 562) optimism were inversely related to resilience endorsement, such that the belief that the condition of the local and national environment would worsen over time was associated with higher resilience endorsement. There was also a significant correlation between local water meaning (average of water importance, water pride, and visits) and resilience endorsement (*r* = 0.186, *N* = 562), such that participants who attached more meaning to their local water source(s) were more likely to endorse resilience principles. Next, a belief in the importance/danger of climate change was significantly correlated with resilience endorsement (*r* = 0.308, *N* = 562), such that individuals who strongly believed that climate change was a critical issue were more likely to endorse resilience principles. Finally, the willingness to accept both a personal (*r* = 0.245, *N* = 562) and societal (*r* = 0.200, *N* = 562) change was correlated with resilience endorsement, such that individuals who were more willing to make a change were more likely to endorse resilience principles.

### Predicting resilience endorsement

A stepwise multiple regression was performed with all demographic, psychological, and environmental variables entered as predictors, and overall resilience endorsement as the criterion, in order to determine which of our measures best predicted endorsement of resilience principles. Overall, the model explained a significant 35.5% of the variance in resilience endorsement, *R* = 0.606; *F*(10, 558) = 31.76, *p* < 0.001. For our demographics measures, age (*sr*^2^ = 0.05), religious importance (*sr*^2^ = 0.01), and political party (*sr*^2^ = 0.01) emerged as significant unique predictors of resilience endorsement. For our psychological measures, openness to experience (*sr*^2^ = 0.04), empathy (*sr*^2^ = 0.03), self-efficacy (*sr*^2^ = 0.01), and internal locus of control (*sr*^2^ = 0.01) were significant unique predictors of resilience endorsement. Finally, for our environmental measures, belief in the importance/danger of climate change (*sr*^2^ = 0.03), local optimism (*sr*^2^ = 0.01), and willingness to accept a personal change (*sr*^2^ = 0.01) were significant unique predictors.

### High/medium/low resilience endorsement

In order to further explore the individual characteristics that relate to resilience endorsement, we divided our participants into 3 groups based on their overall resilience endorsement scores: Low Endorsement (*N* = 187), Medium Endorsement (*N* = 186), and High Endorsement (*N* = 184). We then completed a series of one-way MANOVAs with Scheffe post-hoc tests in order to examine whether our 3 groups differed in terms of demographic makeup, psychological characteristics, and attitudes toward the environment.

#### Demographics

In the overall MANOVA, there was a statistically significant difference in basic demographics as a function of resilience endorsement group, *F*(30, 1082) = 2.40, *p* < 0.001, Wilk’s Λ = 0.879, η_ρ_2 = 0.06. Between-subjects ANOVAs with a Bonferroni correction showed that age, religious attendance, religious importance, and political party all significantly differed as a function of resilience endorsement group (Table 1). Scheffe post-hoc analyses demonstrated that the low resilience endorsement group was significantly younger (*p* = 0.001), attended religious services more regularly (*p* < 0.001), and rated religion as more important (*p* = 0.004), than the high endorsement group, but did not differ from the medium endorsement group (all p’s > 0.05). The low endorsement group was also more politically conservative than either the medium (*p* = 0.009), or the high (*p* = 0.002), groups, whereas the medium and high endorsement groups did not differ. The 3 groups did not significantly differ on any other variables (all p’s > 0.05).

**Table 1:** ANOVA results for the demographic predictors with resilience group as the criterion.

Predictor	Sum of Squares	*df*	Mean Square	*F*	*p*	η_ρ_^2^
Sex	1.53	2	0.76	3.14	0.044	0.011
Age Group	57.14	2	28.57	6.87	0.001	0.024
Country	0.70	2	0.35	1.41	0.245	0.005
Area	0.14	2	0.07	0.15	0.865	0.001
Year in Area	4.98	2	2.49	1.47	0.231	0.005
Highest Education	3.52	2	1.76	0.70	0.499	0.003
Employment Status	4.86	2	2.43	0.33	0.717	0.001
Income	19.49	2	9.75	2.38	0.094	0.009
Marital Status	6.07	2	3.03	4.01	0.019	0.014
Children	0.82	2	0.41	1.79	0.167	0.006
Religious Attendance	32.27	2	16.63	8.02	<0.001	0.028
Religious Importance	15.01	2	7.51	5.76	0.003	0.020
Political Party	7.67	2	3.83	7.35	0.001	0.026

Note: Wilk’s Λ = 0.879, p < 0.001.

#### Psychological factors

In the overall MANOVA, there was a statistically significant difference in scores on our psychological measures as a function of resilience endorsement group, *F*(20, 1096) = 7.38, *p* < 0.001, Wilk’s Λ = 0.777, η_ρ_2 = 0.12. Between-subjects ANOVAs with a Bonferroni correction revealed several differences among the 3 endorsement groups on our psychological measures.

For the personality questionnaire, openness to experience, conscientiousness, neuroticism, and agreeableness all differed as a function of resilience endorsement group (Table 2). Scheffe post-hoc analyses indicated that the low resilience group had significantly lower openness to experience scores than both the medium (*p* < 0.001) and the high (*p* < 0.001) resilience endorsement groups, although the medium and high groups did not differ (p > 0.05). The low endorsement group also had lower conscientiousness scores (*p* = 0.001), and higher neuroticism scores (*p* = 0.003) than the high endorsement group, but did not differ from the medium group. Finally, the low resilience group had significantly lower agreeableness scores than both the medium (*p* < 0.001) and the high *(p* < 0.001) resilience endorsement groups, and the medium resilience endorsement group had lower agreeableness scores than the high resilience endorsement group *(p* < 0.001). Extraversion scores did not differ among the 3 groups.

**Table 2:** ANOVA results for the psychological predictors as a function of resilience group.

Predictor	Sum of Squares	*df*	Mean Square	*F*	*p*	η_ρ_^2^
Openness to Experience	89.23	2	44.62	36.41	<0.001	0.116
Conscientiousness	19.92	2	9.96	7.44	0.001	0.026
Extraversion	3.28	2	1.64	0.76	0.466	0.003
Agreeableness	89.44	2	44.72	32.58	<0.001	0.105
Neuroticism	21.85	2	10.92	6.01	0.003	0.021
Internal Locus of Control	27.08	2	13.54	14.30	<0.001	0.049
External Locus of Control	24.58	2	12.29	13.61	<0.001	0.047
Empathy	559.91	2	279.96	19.44	<0.001	0.065
Self-Efficacy	10.50	2	5.25	12.20	<0.001	0.042
Resistance to Change	8.40	2	4.20	6.52	0.002	0.023

Note: Wilk’s Λ = 0.777, p < 0.001.

Both internal and external locus of control scores also significantly differed as a function of resilience endorsement group. Scheffe post-hoc analyses showed that the low resilience endorsement group had significantly lower internal locus of control scores, and higher external locus of control scores, than both the medium and the high resilience endorsement groups *(*all *p*’s < 0.05). The medium and high groups, however, did not differ on either measure (all *p*’s > 0.05).

Empathy scores significantly differed as a function of resilience endorsement group such that the low endorsement group had significantly lower empathy than the medium or high groups, and the medium group had lower empathy than the high endorsement group (all *p*’s < 0.01).

Self-efficacy scores also significantly differed as a function of resilience endorsement group (Table 2) such that the low endorsement group had significantly lower self-efficacy scores than both the medium (*p* = 0.007) and high (*p* < 0.001) groups, although the medium and high groups did not differ (*p* = 0.22).

Finally, resistance to change scores significantly differed as a function of resilience endorsement group such that the low endorsement group was significantly more resistant to change the high endorsement group (*p* = 0.002). The low endorsement group, however, did not significantly differ from the medium endorsement group, and the medium and high groups did not differ (all *p*’s > 0.05).

#### Environmental factors

In the overall MANOVA, there was a statistically significant difference in scores on the environmental measures as a function of resilience endorsement group, *F*(12, 1102) = 8.55, *p* < 0.001, Wilk’s Λ = 0.837, η_ρ_2 = 0.09. Between-subjects ANOVAs with a Bonferroni correction showed that overall belief in the importance of climate change significantly differed as a function of resilience endorsement group (Table 3), with a Scheffe post-hoc analysis showing that the low resilience endorsement group rated climate change as less important than did the medium (*p* < 0.001) and high (*p* < 0.001) endorsement groups. The medium and high groups, however, did not differ (*p* = 0.33).

**Table 3:** ANOVA results for the environmental predictors with resilience group as the criterion.

Predictor	Sum of Squares	*df*	Mean Square	*F*	*p*	η_ρ_^2^
Importance of Climate Change	29.16	2	14.58	26.99	<0.001	0.088
Local Optimism	7.70	2	3.85	8.06	<0.001	0.028
National Optimism	5.66	2	2.83	4.94	0.007	0.017
Accept Personal Change	24.21	2	12.10	13.82	<0.001	0.047
Accept Non-Personal Change	20.75	2	10.38	12.59	<0.001	0.043
Water Meaning	19.93	2	9.46	7.73	<0.001	0.027

Note: Wilk’s Λ = 0.837, p < 0.001.

Both local and national optimism scores also differed as a function of resilience endorsement group. Scheffe post-hoc analyses showed that the low resilience endorsement group was significantly more optimistic than either the medium or high endorsement groups about the state of the future environment at both the local and national level (all p’s < 0.05), although the medium and high groups did not differ in either local or national optimism.

Next, scores on both the personal and societal “willingness to change” measures differed as a function of resilience endorsement group (Table 3). Scheffe post-hoc analysis showed that for the personal variable, the low endorsement group was less willing to accept a personal change than the high endorsement group (*p* < 0.001), and the medium endorsement group was less willing to accept a personal change than the high endorsement group (*p* = 0.005). However, the low and medium groups did not significantly differ. For the societal change measure, the low endorsement group was significantly less willing to accept a change than either the medium or the high endorsement group (all *p’s* < 0.001). The medium and high groups, however, did not differ.

Lastly, scores on the overall water meaning measure significantly differed as a function of endorsement group, such that the low resilience endorsement group rated their local water source as less personally meaningful than did the high endorsement group (*p* = 0.001). No other relationships were significant.

## Discussion

Resilience building is, above all, a governance issue^13,32,41,42^. Identifying baseline endorsement of water resilience provides a first, critical step in building further support for resilience principles for managing and governing water resources. Important to this argument is that individuals have agency and collectively can effect change at a much larger scale^18,19,43–46^. Also important is the acknowledgement that shifting mindsets of individuals represent one of the best, most effective levers we have for effecting change^22,47,48^.

Despite the increasing number of studies conducted on resilience assessment and measurement^49–52^, little attention has been paid to the extent to which, at the *individual level*, there is endorsement of the concept. Overall, we found considerable societal endorsement of water resilience by the Canadian and US public. It is promising to see that society endorses (i.e., agree with and see the importance of) social-ecological resilience as a framework for managing and governing water resources. In the next two sections, we discuss the key findings from this study and future research directions.

### Individual differences and resilience endorsement predictors

**Figure 2:**
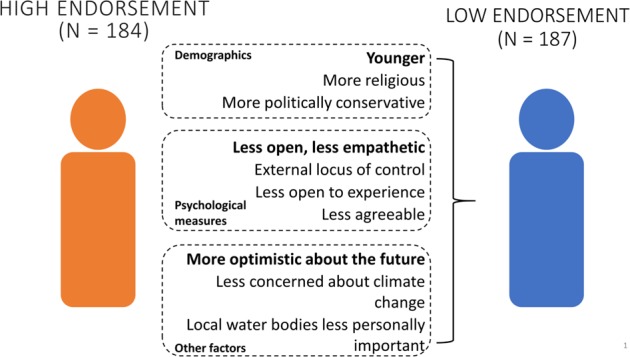
Demographic, psychological and other, environment-focused factors that predict low water resilience endorsement at the individual level. Predictors are ‘bundled’ by the three types of factors, and listed in order of importance, with the best predictor in bold font.

Although overall resilience endorsement was quite high, there were individual variations in endorsement that were associated with differences in a variety of demographic, psychological, and environmental factors. In general, these factors varied between the low and high resilience endorsement groups only, depending on the specific predictor (Fig. 2). Therefore, we focus our discussion on the differences between the low and high endorsement groups.

First, an analysis of the demographic factors revealed a number of characteristics that differed between the high and low resilience endorsement groups. Specifically, the low endorsement group tended to be significantly younger, more religious, and more politically conservative than the high endorsement group. This is partially consistent with past research on demographic predictors of climate change. For example, in the climate change literature, it has been well-established that politically conservative individuals^53–57^, and religious individuals^57,58^, are less likely to engage in pro-environmental behaviours or endorse interventions to mitigate the effects of climate change. However, our finding that younger individuals were less likely to endorse the concept of water resilience is in stark contrast to past literature that has demonstrated that younger individuals are typically *more* likely to support pro-environmental behaviours than older individuals^57,59,60^. This finding may indicate an important deviance between findings from other fields like climate change scepticism, and is worth examining further in future research studies. Interestingly, demographic factors that were expected to be associated with pro-environmental attitudes, such as gender and education^57,60,61^, were not associated with attitudes towards water resilience. One explanation for these disparate findings is that, overall, endorsement for water resilience was quite high with little variability. As such, only those demographic factors that had a strong influence on attitudes differentiated between our high and low endorsement groups. However, this does not explain why there was a significant age difference between our high and low endorsement groups that was in direct contrast to what has been shown in the literature.

With respect to the psychological measures, our findings revealed several significant predictors of resilience endorsement. First, the low endorsement group were less open to experience, less conscientious, and less agreeable as compared to the high endorsement group. These findings replicate those that have shown similar relationships between pro-environmental attitudes and openness to experience, agreeableness, and conscientiousness^62,63^. Interestingly, we found a negative relationship between resilience endorsement and neuroticism, such that individuals higher in neuroticism were *less* likely to endorse a resilience perspective. This is in contrast to Hirsch^62^, who found a positive, albeit small, relationship between environmental engagement and neuroticism. However, Milfont and Sibley^63^ found that neuroticism was both negatively (study 1 and 3) and positively (study 2) associated with environmental engagement. One explanation for the current findings could be that individuals high in neuroticism may express environmental concerns (consistent with their tendency to worry excessively), but may be less willing to endorse radical solutions to these concerns, especially if the outcome is uncertain. Further experimentation is necessary in order to determine the association between neuroticism and attitudes towards pro-environment interventions.

In addition to personality, we also found a positive association between resilience endorsement and empathy, such that individuals from the high endorsement group had higher empathy scores. Empathy is correlated with both agreeableness and openness to experience^64^, thus it follows that the high endorsement group would show high levels of all three traits. This finding is consistent with Brown *et al*.^21^, who recently demonstrated that a lack of empathy limits motivations to conserve the environment and enhance sustainability. It is also consistent with the findings of Berenguer^65^, who showed that individuals who were induced into a low empathy state were less likely to engage in helping behaviour toward the environment as compared to individuals induced into a high empathy state.

Next, the low endorsement group had a significantly higher external locus of control, and a significantly lower internal locus of control, than the high endorsement group. In other words, the low endorsement group was more likely to believe that they have little control over the events in their life (e.g., successes and failures are due to external factors such as luck), whereas the high endorsement group was more likely to believe that they have power over the events in their life (e.g., successes and failures are due their own efforts and abilities). With respect to resilience endorsement, this finding suggests that those who have an external locus of control are less likely to believe that taking a resilience perspective to tackle water-related problems will make a positive difference. Previous research has shown a similar pattern, such that individuals with an external locus of control are less likely to engage and support measures to mitigate the negative effects of climate change^66,67^.

Similarly, the low endorsement group also had lower self-efficacy scores than the high endorsement group. Self-efficacy is the belief that one has the ability to succeed in a given action^68^, thus it follows that individuals who score lower on this trait would be less willing to endorse an action or perspective that could change the way in which we manage our water resources. Gifford^66^ notes that individuals low in self-efficacy have a sense of fatalism, such that they do not believe that any individual action can have an impact on the environment, and thus are less likely to engage in collective action.

With respect to our environmental factors, we found four significant predictors of resilience endorsement. First, although both groups rated climate change as an important issue, the low resilience endorsement group rated climate change as less important than the medium and high endorsement groups. As such, it follows that if individuals do not believe that there is a serious problem in the first place, they are less likely to endorse a new water management perspective that is designed to mitigate the effects of this problem.

Second, the low resilience endorsement group was significantly more optimistic than the high endorsement group. about the state of the future environment at both the local and national level These findings are consistent with the climate change literature, which shows that climate change sceptics are more hopeful and optimistic about the future state of the environment^53^ than individuals who acknowledge the dangers of climate change.

Third, the low endorsement group rated their local water sources as less personally meaningful than the high endorsement group. Research on “sense of place” has shown that individuals with lower place attachment are less likely to support and engage in pro-environmental behaviours^66,69^, thus it is unsurprising that individuals who are less attached to their local water sources are also less interested in supporting measures to protect and manage their local water source.

Finally, the low resilience endorsement group was less willing to accept personal or societal change as compared to the high endorsement group. This may be associated with their low self-efficacy scores and their high external locus of control scores, such that they are less willing to engage in collective action.

This research presents a novel context in which to test predictive individual difference variables. As such, there are some limitations to our study. First, statements developed to assess resilience endorsement simplified the complexity of the principles. This was necessary for pragmatic reasons to maintain a questionnaire of reasonable length and thus required the researchers to distill and assess one or two key factors of focus for each principle.

Second, the statements provided to respondents were broadly about ‘water’ and did not endeavour to distinguish among the various ways in which water is used and present on the landscape. Accordingly, the assessment is coarse, and provides an opportunity for deeper inquiries into these areas.

Third, respondents were selected via a third-party data provider (MTurk), thus there is the potential for responses to be biased as a result. For example, MTurk participants tend to complete surveys for unusually low pay, have the ability to pick and choose which projects they wish to complete based on their interests and experience (e.g., they might only select surveys that are similar to those they have completed in the past), and often discuss surveys and/or researchers on online forums, thus potentially limiting the representativeness of the sample. Interestingly, however, a recent study by Paolacci and Chandler^34^ demonstrated that the data from MTurk samples are typically similar in quality and reliability to traditional lab-based samples, and the samples themselves are typically more heterogenous in nature, indicating that any biases introduced by using a third-party provider may be minimal. Nonetheless, we engaged in a series of best practices to minimize potential response biases (e.g., using strict qualification criteria to screen out poor-performing workers; using clear language), but acknowledge that they may still have influenced the results.

Finally, we recognize that by not defining ‘water’ to the respondents, their own preconceptions may have influenced the results. We return to the diversity of ways in which water resilience has been described (e.g., Falkenmark *et al*.^70^ define water in terms of ‘blue’ and ‘green’ including evaporation and transpiration), and note that we did not include statements in our scale that addressed this conceptualization of water, but focused primarily on ‘visible’ water. Considering water from a more complete perspective offers an important avenue for further study.

Much has been written about individual differences in relation to other questions, particularly with respect to attitudes towards climate change, and our findings are largely consistent with that body of literature, with the exception of the age effect. However, this study represents a distinct line of inquiry and no a priori assumptions were made about alignment between climate change studies and our focus on water resilience. With this baseline understanding of the individual difference factors that predict attitudes towards water resilience, we can begin to use this information, and benefit from studies in related fields such as climate change perceptions, to create targeted interventions to change attitudes towards resilience. For example, by empowering individuals with low self-efficacy so that they feel more comfortable taking action on environmental issues, by increasing the place attachment of individuals with low sense of place, or by creating messaging that is relevant to religious or political organizations to help change perspectives, we may see an increase in pro-environmental action. Research on climate change scepticism has already demonstrated the potential benefits of taking an individualized approach^71^, thus it is likely that using similar targeted messaging to enhance resilience endorsement will effect positive change. It is worth emphasizing that while the predictors and potential approaches may be similar between water resilience and other issues like climate change scepticism, the issues themselves are distinct.

## Conclusions

The findings from our efforts to operationalize the seven social-ecological resilience principles in the context of water resources (i.e., via a ‘water resilience scale’) highlight the current state of endorsement of water resilience for the Canadian and US public. Endorsement was, on average, high but with sufficient variability that we identified three water resilience endorsement groups: a low, medium, and high endorsement group. Ultimately, we found that a number of key demographic, psychological, and environmental factors differed amongst the three resilience endorsements groups (e.g., the low endorsers significantly differed from the high-endorsers with respect to religiosity, political leaning, personality, and self-efficacy, among others), demonstrating that individual differences are associated with water resilience endorsement. We see the development of the water resilience scale and these findings as a first, baseline assessment. Future research should consider incorporating further nuances of endorsement and consider approaches to motivate positive changes in endorsement levels, using the predictor variables as a basis for engaging with these individuals to increase endorsement.

## Supplementary information

Supplementary Information.

## Data Availability

The aggregated, deidentified dataset analysed during the current study is available from the corresponding author upon request.
