# Supplementary material for: Individual differences predict endorsement of water resilience
Source: Sci Rep. 2020 Apr 6;10:5974. doi: 10.1038/s41598-020-62896-x (PMC7136210; doi:10.1038/s41598-020-62896-x)
Supplement: Supplementary file 1 — Supplementary Information. [file 41598_2020_62896_MOESM1_ESM.pdf]

Supplementary Information

Julia Baird[1,2]*, Gillian Dale[1], and Sherman Farhad[1]

[1]Environmental Sustainability Research Centre, Brock University, Ontario, Canada

[2]Department of Geography and Tourism Studies, Brock University, Ontario Canada

*Corresponding author: jbaird@brocku.ca

Table S1. Demographics for all participants, and for the low, medium, and high resilience endorsement groups.

		All	Low Group	Medium Group	High Group
**Sex**					
	Male	241	73	72	94
	Female	321	114	115	92
**Age Group**					
	18-24	93	38	34	21
	25-29	128	56	36	36
	30-34	118	40	38	40
	35-39	78	20	29	28
	40-44	54	6	20	27
	45-49	32	12	11	9
	50+	56	15	18	23
**Country**					
	Canada	268	93	95	79
	USA	294	94	92	107
**Area**					
	Urban	239	80	76	83
	Suburban	242	77	85	78
	Rural	81	30	26	25
**Years in Area**					
	Less than 1	39	14	14	11
	1-5	195	55	72	67
	6-9	94	32	31	31
	10+	234	86	70	77
**Highest Education**					
	High School	69	27	24	18
	Vocational/Tech	14	5	6	3
	Some College	134	45	46	43
	Associate Degree	59	14	17	28
	Bachelors	222	71	77	72
	Masters	52	17	17	18
	PhD	8	5	0	3
	Other	4	3	0	1
**Employment Status**					
	Full-Time	308	103	97	106
	Part-Time	45	15	16	14
	Self-Employed	71	21	27	23
	Unemployed/Student	93	29	35	29
	Other	45	19	12	14
**Income**					
	Under $20,000	67	23	29	15
	$20,000-39,999	154	61	46	47
	$40,000-74,999	186	57	60	69
	$75,000-99,999	77	25	27	24
	$100,000+	78	21	25	31
**Marital Status**					
	Single/Never Married	302	110	108	84
	Married/Common Law	232	71	69	90
	Other	28	6	10	12
**Children**					
	Yes	200	69	57	73
	No	362	118	130	113
**Religious Attendance**					
	Never	289	85	93	109
	Seldom	113	35	42	36
	Once/year	59	18	22	19
	Once or twice/month	37	15	11	11
	At least once/week	64	34	19	11
**Religious Importance**					
	Not at all important	279	74	98	106
	Not too important	82	33	23	25
	Somewhat important	114	43	36	35
	Very important	87	37	30	20
**Political Party**					
	Conservative/Republican	94	47	23	24
	Liberal/Democrat	225	74	79	70
	Other	243	66	85	92

Table S2. Means and standard deviations for psychological measures for all participants, and low, medium, and high resilience endorsement groups.

	**All**<br>*M (SD)*	**Low Group**<br>*M (SD)*	**Medium Group**<br>*M (SD)*	**High Group**<br>*M (SD)*
Openness to Experience	5.36 (1.18)	4.81 (1.18)	5.51 (1.04)	5.75 (1.10)
Conscientiousness	4.94 (1.17)	4.70 (1.13)	4.98 (1.16)	5.16 (1.18)
Extraversion	3.56 (1.47)	3.59 (1.32)	3.45 (1.48)	3.62 (1.59)
Agreeableness	5.14 (1.24)	4.65 (1.18)	5.14 (1.22)	5.63 (1.11)
Neuroticism	3.31 (1.36)	3.56 (1.24)	3.28 (1.31)	3. 08 (1.49)
Internal Locus of Control	5.36 (1.00)	5.06 (1.09)	5.47 (0.86)	5.57 (0.95)
External Locus of Control	3.40 (0.97)	3.66 (1.06)	3.37 (0.79)	3.15 (0.98)
Empathy	12.99 (3.91)	11.77 (3.93)	12.97 (3.81)	14.22 (3.63)
Self-Efficacy	3.86 (0.68)	3.67 (0.66)	3.89 (0.64)	4.01 (0.67)
Resistance to Change	3.54 (0.81)	3.67 (0.75)	3.58 (0.81)	3.38 (0.84)

Table S3. Means and standard deviations for environmental measures for all participants, and low, medium, and high resilience endorsement groups.

	**All**<br>*M (SD)*	**Low Group**<br>*M (SD)*	**Medium Group**<br>*M (SD)*	**High Group**<br>*M (SD)*
Importance of Climate Change	4.52 (.77)	4.20 (0.88)	4.62 (0.70)	4.73 (0.60)
Local Optimism	-0.77 (0.70)	-0.61 (0.69)	-0.82 (0.67)	-0.89 (0.71)
National Optimism	-0.74 (0.76)	-0.60 (0.74)	-0.80 (0.72)	-0.83 (0.82)
Accept Personal Change	3.99 (0.96)	3.76 (1.02)	3.95 (0.94)	4.26 (0.84)
Accept Non-personal Change	3.95 (0.93)	3.69 (0.95)	4.04 (0.90)	4.13 (0.87)
Water Meaning	3.99 (1.12)	3.78 (1.14)	3.95 (1.11)	4.22 (1.06)

Supplementary Methods: Water Resilience Scale

The following questions will ask about your opinion on the importance of several factors related to water resilience and management.

Scale: 1 = very unimportant, 2 = somewhat unimportant; 3 = neither important nor unimportant; 4 = somewhat important; 5 = very important

1. How important is it to you that many perspectives are included in decision making about water (e.g., science, government, community, traditional)?
2. How important is it to you that a range of groups are meaningfully included in decision making about water (e.g., they are involved to a greater extent than just providing advice or information to authorities)?
3. How important is it to you that water bodies are able to support many different types of fish and water organisms?
4. How important is it to you that there is communication with stakeholders (e.g., people who live and work near a water source) when making decisions about a water body, even if they might not hold any authority to make decisions?
5. How important is it to you that potential barriers to the movement of fish and other water organisms within and between water bodies factors into decision making (e.g., ensuring fish habitat spans an entire river)?
6. How important is it to you that decision making about water considers long term changes and impacts (e.g., an increase in water pollution can cause large algae blooms over time)?
7. How important is it to you that social issues like population growth or urbanization are considered when making decisions about water?
8. How important is it to you that there is more than one entity or agency in charge of making decisions about water resources (that decision making is distributed among several groups/agencies)?
9. How important is it to you that those who manage and make decisions about water resources are open to trying new approaches (e.g., small experiments using untested ideas), and learning and adapting from those approaches?
10. How important is it to you that when decisions are made about water that other factors that might influence the water are considered (e.g., policy makers consider all users and uses of a water body when revising or making new policies about it)?

For the following questions, please indicate the extent to which you agree with each statement:

Scale: 1 = strongly disagree, 2 = somewhat disagree; 3 = neither agree nor disagree; 4 = somewhat agree; 5 = strongly agree

11. Decision makers should focus on immediate threats to our water, and not spend a lot of time on monitoring for longer term potential problems.
12. I am not concerned about what fish or other organisms are living in the water.

13. Better decisions are made about water when there are opportunities for groups other than the government to influence those decisions.
14. I am not concerned about how well fish and other water organisms can move through water bodies.
15. I want decision makers to use established ways of managing water rather than try new ways that might not work.
16. I am not concerned about having input into decisions about water bodies close to me.
17. Better decisions are made about water when many sources of information are used.
18. It is worthwhile to spend resources (money, time, build infrastructure) to prepare for possible risks to water, even if potential problems might never occur.

For the last question: *Scale: 1 = No, 2 = Yes; 3 = Unsure*

19. Related to the above question, would it be worthwhile to spend resources to prepare for possible risks if they could help manage a whole range of possible issues, rather than one specific risk?
